# Magnetic-Resonance-Imaging-Based Left Atrial Strain and Left Atrial Strain Rate as Diagnostic Parameters in Cardiac Amyloidosis

**DOI:** 10.3390/jcm11113150

**Published:** 2022-06-01

**Authors:** Vanessa Sciacca, Jan Eckstein, Hermann Körperich, Thomas Fink, Leonard Bergau, Mustapha El Hamriti, Guram Imnadze, Denise Guckel, Henrik Fox, Muhammed Gerçek, Martin Farr, Wolfgang Burchert, Philipp Sommer, Christian Sohns, Misagh Piran

**Affiliations:** 1 Clinic for Electrophysiology, Herz-und Diabeteszentrum NRW, Ruhr-Universität Bochum, 32545 Bad Oeynhausen, Germany; tfink@hdz-nrw.de (T.F.); lbergau@hdz-nrw.de (L.B.); melhamriti@hdz-nrw.de (M.E.H.); gimnadze@hdz-nrw.de (G.I.); dguckel@hdz-nrw.de (D.G.); psommer@hdz-nrw.de (P.S.); 2 Institute for Radiology, Nuclear Medicine and Molecular Imaging, Herz-und Diabeteszentrum NRW, Ruhr-Universität Bochum, 32545 Bad Oeynhausen, Germany; jeckstein@hdz-nrw.de (J.E.); hkoerperich@hdz-nrw.de (H.K.); wburchert@hdz-nrw.de (W.B.); mpiran@hdz-nrw.de (M.P.); 3 Clinic for Thoracic and Cardiovascular Surgery, Herz-und Diabeteszentrum NRW, Ruhr-Universität Bochum, 32545 Bad Oeynhausen, Germany; hfox@hdz-nrw.de; 4 Heart Failure Department, Herz-und Diabeteszentrum NRW, Ruhr-Universität Bochum, 32545 Bad Oeynhausen, Germany; 5 Clinic for General and Interventional Cardiology/Angiology, Herz-und Diabeteszentrum, 32545 Bad Oeynhausen, Germany; mugercek@hdz-nrw.de (M.G.); mfarr@hdz-nrw.de (M.F.)

**Keywords:** amyloidosis, left atrial strain, left atrial function, left atrial strain rate, cardiac imaging

## Abstract

**Aims:** The present study aims to evaluate magnetic-resonance-imaging (MRI)-assessed left atrial strain (LAS) and left atrial strain rate (LASR) as potential parameters for the diagnosis of cardiac amyloidosis (CA), the distinction of clinical subtypes and differentiation from other cardiomyopathies. **Methods and results:** LAS and LASR were assessed by MRI feature tracking in patients with biopsy-proven CA. LAS and LASR of patients with CA were compared to healthy subjects and patients with hypertrophic cardiomyopathy. LAS and LASR were also analyzed concerning differences between patients with transthyretin (ATTR) and light chain amyloidosis (AL). A total of 44 patients with biopsy-proven CA, 19 patients with hypertrophic cardiomyopathy and 24 healthy subjects were included. In 22 CA patients (50%), histological examination identified ATTR as CA subtype and AL in the remaining patients. No significant difference was observed for reservoir, conduit or booster LAS in patients with AL or ATTR. Reservoir LAS, conduit LAS and booster LAS were significantly reduced in patients with CA and HCM as compared to healthy subjects (*p* < 0.001). Reservoir LAS and booster LAS were significantly reduced in CA as compared to HCM patients (*p* < 0.001). A linear correlation was observed between LA global reservoir strain and LA-EF (*p* < 0.001, r = 0.5), conduit strain and global longitudinal LV strain (*p* < 0.001, r = 0.5), global booster strain rate and LA-EF (*p* < 0.001, r = 0.6) and between global booster strain rate and LA area at LVED (*p* < 0.0001, 0.5). **Conclusions:** LAS and LASR are severely impaired in patients with CA. The MRI-based assessment of LAS and LASR might allow non-invasive diagnosis and categorization of CA and its distinct differentiation from other hypertrophic phenotypes.

## 1. Introduction

Cardiac amyloidosis (CA) is an infiltrative disease of the myocardium caused by the interstitial deposition of insoluble amyloid fibrils [1]. The misfolding of >30 proteins with an unstable tertiary structure has been identified as the mechanism of formation of amyloid fibrils [2]. The vast majority of clinical apparent cases of CA is caused by either transthyretin amyloidosis (ATTR) or light chain amyloidosis (AL). By means of molecular genetics, ATTR could further be distinguished in a non-hereditary wild type of amyloidosis (ATTRwt) and a hereditary variant (ATTRmt) [3]. Irrespective of the underlying subtype of CA, a notable hypertrophic phenotype develops, resulting in a severe restrictive cardiomyopathy with diastolic dysfunction. Mortality in patients with CA is high with survival rates of 4–5 years in patients with ATTR amyloidosis and less than 6 months in patients with untreated AL [4]. The early and definite diagnosis of CA is therefore crucial in order to initiate adequate therapy and improve clinical outcome. Transthoracic echocardiography is a broadly available imaging technique that can detect features rising suspicion of CA, such as left ventricular wall thickening, biatrial enlargement, thickened valves, elevated right ventricular systolic pressure, sparkling appearance of a granulated myocardial wall, pericardial effusion, or restrictive transmitral filling patterns. Cardiac MRI, on the other hand, can show detailed information on systolic function and cardiac structure. The advantage of cardiac MRI is its unique ability to enable tissue characterization, allowing it to differentiate amyloidosis from nonamyloid wall-thickening disorders. Myocardial scintigraphy with bone-avid tracers has a high sensitivity and specificity for ATTR and may be helpful in the diagnostic process. However, endocardial biopsy is still required in a relevant number of patients for the definite diagnosis and differentiation of CA. Therefore, novel diagnostic tools are required to further optimize non-invasive diagnosis and the differentiation of CA and other phenotypic mimics. The diagnosis of CA is complex and multimodal so that the disease is still understood as underdiagnosed [5]. Diagnosis is often deferred due to a lack of disease awareness and difficulties in disease recognition as to the heterogeneity of symptoms at presentation [6,7]. Hence, new tools are needed for the diagnosis of CA and its differentiation from other phenotype mimics. Although amyloid deposition may affect every chamber of the heart, recent investigations focus on the impact of amyloid deposition on the left ventricular wall architecture and function due to the typical clinical appearance with wall thickening, diastolic and systolic heart failure and low cardiac output [8,9]. In spite of recently impressive findings in terms of high-throughput precision phenotyping of left ventricular hypertrophy by cardiovascular deep learning algorithms, the left atrium has become a target of interest in patients with CA. [10]. Nevertheless, most studies analyzing the role of the left atrium in CA merely set left atrial dimensions as surrogate parameters of disease activity, severity and chronicity without taking into account left atrial function. Impaired left atrial function has been identified as a significant driver of global cardiac dysfunction and as a distinct clinical entity presenting as left atrial failure [11]. Left atrial strain (LAS) and left atrial strain rate (LASR) measured during reservoir function at left ventricular systole and isovolumetric relaxation or during conduit function at left ventricular diastole or during booster function at active atrial contraction are known specific parameters of left atrial function with known prognostic relevance [12]. Nowadays, only sparse data on MRI-based LAS and LASR measurements in patients with CA is available. The great potential of MRI-based left atrial functional assessment lies in early disease detection, potential non-invasive diagnosis and phenotypic differentiation.

The aim of this study is to assess LAS and LASR using MRI in a cohort of patients with biopsy-proven CA and to compare these results to a healthy cohort for the demonstration of the effect of CA on the left atrial function. Furthermore, the LAS and LASR values were compared to a cohort of patients with diagnosed hypertrophic cardiomyopathy in order to evaluate the usefulness of left atrial function parameters as markers for the differentiation of CA in the context of diseases with a similar phenotype.

## 2. Methods

### 2.1. Study Population

The present study is a single center observational study on patients with histologically proven CA who underwent cardiac MRI at our institution. Sinus rhythm at the time of cardiac MRI was mandatory for inclusion into the study. Cardiac MRI was performed in all patients undergoing diagnostic work-up because of left ventricular hypertrophy as clinical routine.

### 2.2. Hypertrophic Cardiomyopathy Population

A group of patients with known hypertrophic cardiomyopathy who underwent cardiac MRI during diagnostic work-up served as a control group. Atrial arrhythmia at the time of cardiac MRI was an exclusion criterium.

### 2.3. Healthy Control Group

A group of healthy subjects who voluntarily underwent cardiac MRI at our institution served as a second control group. All subjects gave written informed consent to participate in the study. The individuals within this group were examined by means of a general health assessment questionnaire before being included in the study. The subjects of the control group were fitted by age to patients with CA. Subjects were excluded if cardiovascular diseases, prior cardiovascular surgery, continuous medication for cardiovascular or metabolic disorders, known cardiovascular risk factors or general contraindications to cardiac MRI were present. In case of cardiac MRI revealing myocardial or vessel abnormalities or any other sign of underlying cardiomyopathy, subjects were excluded from this study.

### 2.4. Cardiac MRI

All patients underwent cardiac MRI using a 3.0 Tesla multi-transmit MRI system (Achieva, Philips Healthcare, Best, The Netherlands; Release 5.3.1 and 5.6.1) incorporating dStream technology. Patients were generally examined in the supine position. A vector electrocardiogram was applied in all patients for the acquisition of cardiac triggering. The maximum gradient performance was 40 mT/m with a slew rate of 200 mT/m/ms. A cardiac phased-array coil was used for signal reception. The institutional protocol implemented 2-chamber and 4-chamber long axis views. A stack of an axially acquired stack covering the whole heart as well as a short-axis stack covering the entire left and right ventricles (12–16 slices, no gap) was utilized with cine steady-state free-precession acquisitions (TR/TE/flip angle = 2.7 ms/1.35 ms/42°) for the assessment of cardiac function, morphology and strain. Within one cardiac cycle, 28–45 heart frames were acquired. At a typical heart rate of 70 beats/min, the temporal resolution was 34 or 19 ms in one cardiac phase. Spatial resolution was 1.5 × 1.5 × 8 mm^3^.

### 2.5. Strain Analysis

**Figure 1:**
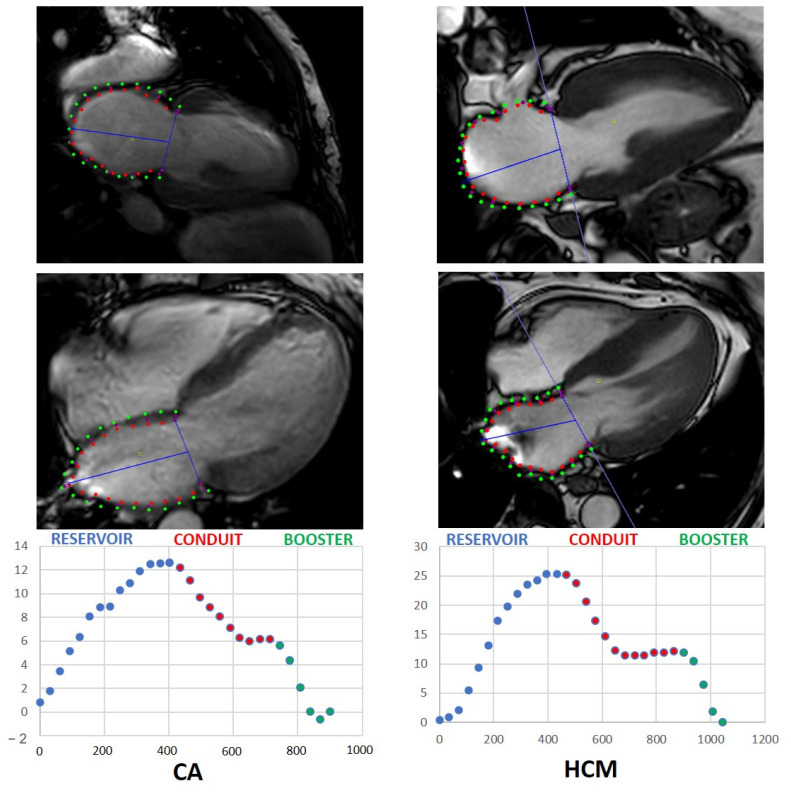
Left atrial strain measurements in patients with CA and HCM. (**Upper row**): longitudinal two-chamber view; (**Lower row**): left atrial longitudinal strain-to-time curve. Red dots in MRI images represent endocardial contours; green dots represent epicardial contours. CA: cardiac amyloidosis; HCM: hypertrophic cardiomyopathy.

The longitudinal axis served as the quantitative parameter for LA strain assessment. The longitudinal strain was expressed in negative values. Strain analysis was conducted using the CVI42^®^ software package (Circle Cardiovascular Imaging Inc., Calgary, AB, Canada, Release 5.12.1) based on cine steady-state free-precession acquisitions. Contours of left atrial endo- and epicard were delineated manually in 2-chamber and 4-chamber long-axis slices. The linings excluded the pulmonary vein ostia as well as the left atrial appendage. The global longitudinal strain (GLS) as well as an individual global longitudinal strain for 4-chamber and 2-chamber views were evaluated. Global longitudinal LAS and global longitudinal LA-SR were estimated for patients with CA and HCM and healthy subjects. LA performance was analyzed as LAS and LA-SR, including reservoir, conduit and booster function for each patient cohort. LA volumetric measures were obtained at the end of diastole and systole by defining the endocardial contours in the axially acquired cine stack. Figure 1 illustrates the different phases of left atrial strain measurement in patients with CA or HCM.

### 2.6. Statistics

Statistical analysis was performed using SPSS. Data are expressed as mean ± standard deviation for continuous variables or as median with interquartile ranges for categorical variables. The baseline characteristics of ATTR and AL patients were compared to each other using a standard t-test. The significance of differences in LA global longitudinal strain (reservoir, conduit and booster) and in LA global longitudinal strain rate (reservoir, conduit and booster) between the cohorts of patients with amyloidosis, those patients with HCM and the healthy subjects was calculated using the non-parametric Kruskal–Wallis test. Correction for multiple testing was not conducted. A correlation analysis was performed to assess possible associations of LA global longitudinal strain and strain rate (reservoir, conduit and booster) to other established parameters. Correlations were investigated using Pearson’s correlation coefficient. *p*-values of <0.05 were considered statistically significant.

## 3. Results

### 3.1. Patient Baseline Characteristics

Forty-four CA patients were enrolled in this study. Thirty-three patients (75%) were male. Mean patient age was 73.7 ± 8.9 years. Mean BMI was 25.2 ± 3.5 kg/m^2^ and mean body surface was 1.9 ± 0.2 m^2^. Mean left ventricular ejection fraction (LV-EF) of the CA population was 46.4 ± 9.6%. The majority of patients (81.1%) suffered from symptomatic heart failure NYHA class III. A small number of patients experienced heart failure at NYHA class II (13.6%) and NYHA class IV (4.5%). Details on cardiovascular comorbidities are summarized in Table 1.

### 3.2. Control Group Baseline Characteristics

Two control groups consisting of 19 patients with hypertrophic cardiomyopathy and 47 healthy subjects were implemented in the statistical analysis. Baseline characteristics of all groups were collected (Table 1, Table 2 and Table 3).

### 3.3. Amyloidosis-Specific Characteristics

Myocardial biopsy was performed in all 44 patients (100%) with histological confirmation of CA. In 22 patients (50%), histological examination identified ATTR as CA subtype, while in the other 22 patients (50%), AL was revealed. The quantitative histological grading of amyloid deposition was assessed, showing that the majority of patients suffered from high-grade amyloid deposition (Table 4).

### 3.4. Left Atrial Strain and Strain Rate in Patients with ATTR and AL

In patients with ATTR, mean reservoir LAS was 7.9 ± 5.9%, mean conduit LAS 5.5 ± 2.9% and mean booster LAS 2.5 ± 4.9%. No significant difference was found between the two subtypes ATTR and AL in terms of reservoir, conduit or booster LAS (*p* = 0.637, *p* = 0.988 and *p* = 0.585, respectively). In patients with ATTR, mean reservoir LASR was −0.5 ± 0.5, mean conduit LASR −0.3 ± 0.3% and mean booster LASR −0.4 ± 0.4% (*p* = 0.697, *p* =0.051 and *p* = 0.104; Table 5).

### 3.5. Functional LA Parameters in Patients with CA and HCM and Healthy Subjects

**Figure 2:**
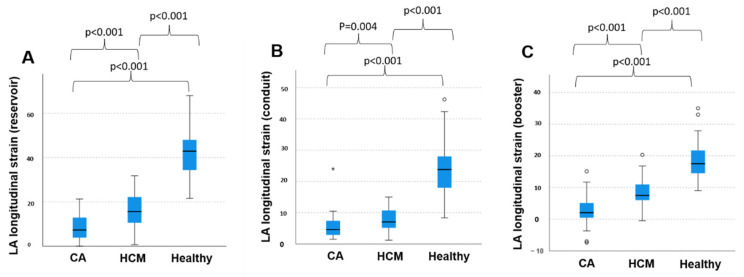
Left atrial strain in patients with cardiac amyloidosis and hypertrophic cardiomyopathy and healthy subjects. (**A**) Longitudinal left atrial reservoir strain, (**B**) longitudinal left atrial conduit strain and (**C**) longitudinal left atrial booster strain within the three groups, respectively. CA: cardiac amyloidosis; HCM: hypertrophic cardiomyopathy; LA: left atrial. *: extreme values, ○: outliers.

**Figure 3:**
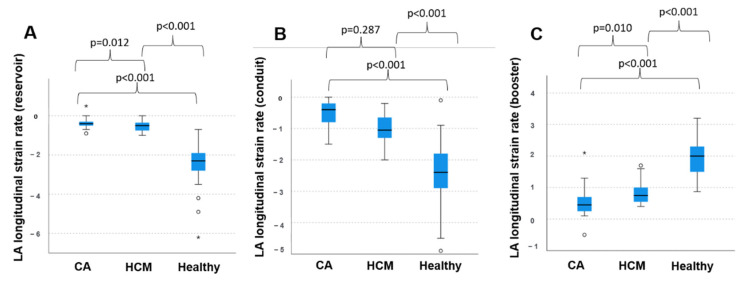
Left atrial strain rate in patients with cardiac amyloidosis and hypertrophic cardiomyopathy and healthy subjects. (**A**) Longitudinal left atrial reservoir strain rate, (**B**) longitudinal left atrial conduit strain rate and (**C**) longitudinal left atrial booster strain rate within the three groups, respectively. CA: cardiac amyloidosis; HCM: hypertrophic cardiomyopathy; LA: left atrial. *: extreme values, ○: outliers.

The mean reservoir LAS was 8.3 ± 5.6% in patients with CA, regardless of the individual amyloid subtype. The mean conduit and booster LAS were 5.5 ± 4.0% and 2.8 ± 4.6% in this group, respectively. In patients with HCM, mean reservoir LAS was 16.8 ± 8.3%. Mean conduit and booster LAS were 7.9 ± 4.0% and 8.9 ± 5.0%, respectively, in this cohort. In healthy patients, mean LAS for reservoir function was 42.0 ± 11.5% and mean conduit and booster were LAS 24.0 ± 8.6% and 18.3 ± 6.0%, respectively. Statistical testing by non-parametric Kruskal–Wallis test found significant differences between all three groups regarding reservoir, conduit and booster LAS (Figure 2). Statistically significant differences were also found between all groups regarding reservoir LASR and booster LASR as well as a significant difference in conduit LASR between healthy subject and patients with HCM and between healthy subjects and patients with CA. No significant difference could be discriminated focusing on booster LASR when comparing patients with CA to patients with HCM (Figure 3).

### 3.6. Correlation of LAS in CA with Established Parameters

**Figure 4:**
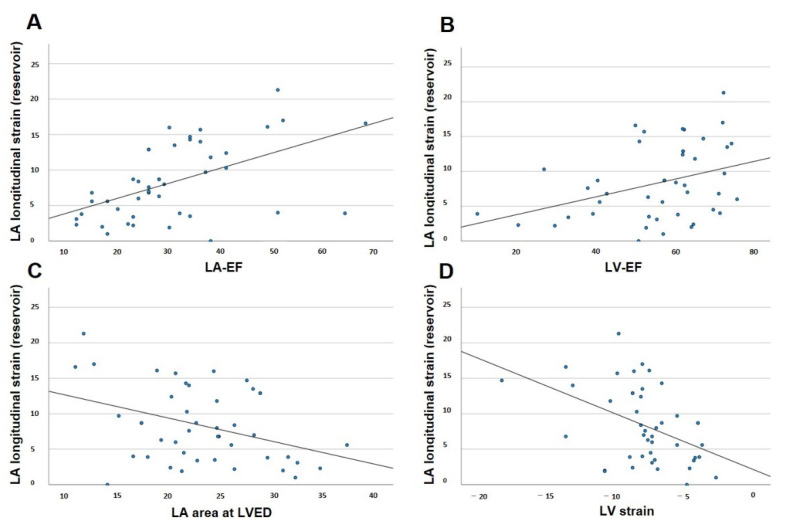
Pearson’s correlation for left atrial reservoir strain and disease-relevant factors in cardiac amyloidosis. (**A**) Correlation of left atrial reservoir strain and left atrial ejection fraction. (**B**) Correlation of left atrial reservoir strain and left ventricular ejection fraction. (**C**) Correlation of left atrial reservoir strain and end-diastolic left atrial area. (**D**) Correlation of left atrial reservoir strain and left ventricular strain. LA-EF: left atrial ejection fraction; LV-EF: left ventricular ejection fraction; LA: left atrial; LV strain: left ventricular global longitudinal strain; LVED: left ventricular end diastole.

**Figure 5:**
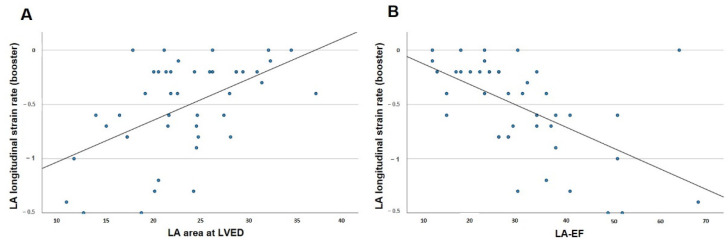
Pearson’s correlation for left atrial booster strain rate and disease-relevant factors in cardiac amyloidosis. (**A**) Correlation of left atrial booster strain rate and end-diastolic left atrial area. (**B**) Correlation of left atrial booster strain rate and left atrial ejection fraction. LA-EF: left atrial ejection fraction; LV-EF: left ventricular ejection fraction; LA: left atrial; LV: left ventricular; LVED: left ventricular end diastole.

The possible linear correlations of reservoir, conduit and booster LAS with the established prognostic parameters in patients with CA were tested as described above. The parameters tested for possible correlation with reservoir, conduit and booster LAS were LV-EF, LV strain, LA-EF and LA area at LVED (Figure 4 and Table 6). These parameters were also tested for a possible correlation with reservoir, conduit and booster LASR (Figure 5). Strong linear correlations with correlation coefficients > 0.5 were observed between global reservoir strain and LA-EF (*p* < 0.001) and between global conduit strain and LV strain (*p* < 0.001). Regarding LASR, strong linear correlations with correlation coefficients >0.5 were observed between global booster strain rate and LA-EF (*p* < 0.001) and between global booster strain rate and LA area at LVED (*p* < 0.0001).

## 4. Discussion

To our best knowledge, the present study is the first to conduct systematic analysis of LAS and LASR assessed by MRI in patients with CA. In addition, this is the first study reporting information on MRI-based LAS and LASR as potential novel parameters for the distinct and non-invasive differentiation between CA and HCM. The main findings of the study are:

1. MRI-assessed LAS as well as LASR are significantly impaired in patients with CA compared to healthy subjects.
2. MRI-assessed LAS and LASR significantly differ in patients with CA and HCM with a significantly stronger decrease in patients with CA.
3. Reservoir LAS and conduit LAS correlate with LA-EF and LV strain, respectively. Booster LASR correlates with LA-EF and LA end diastolic area.

### 4.1. Diagnostic Value of MRI-Based Left Atrial Strain Analysis in Cardiac Amyloidosis

LA function has been studied in detail mainly in echocardiography. In particular, strain analysis based on speckle tracking for direct measurement of intrinsic left atrial myocardial deformation is widely performed for the echocardiographic analysis of LA function, but its clinical value is constricted [13]. The MRI-guided LA functional analysis in patients with CA has mainly focused on the quantification of late gadolinium enhancement and associated alterations of LA-EF to date, while the MRI-guided examination of LAS has not been investigated in patients with amyloidosis yet [14,15]. The present study shows that severe LAS and LASR impairment is frequently present in CA patients compared to healthy subjects, regardless of the CA subtype. This finding might reflect the amount of previously demonstrated extensive LA amyloid depositions in patients with CA [16]. Furthermore, this finding suggests a high diagnostic sensitivity for CA in patients with undefined etiology of myocardial hypertrophy. This observation is in line with previously published studies on LAS and LASR assessed by echocardiography in patients with CA [17]. MRI-based atrial strain analysis might therefore be favorable especially in patients with low echocardiographic image quality due to anatomical issues. The clear differentiation of ATTR and AL from non-invasive assessment remains challenging. In our study, we found no significant difference between patients with ATTR and AL. Our data show that alternative parameters have to be identified to allow for a distinct characterization of ATTR and AL under routine clinical conditions.

In our study, LAS and LASR showed a significant correlation to global longitudinal LV strain, which is an established sensitive predictor of left ventricular dysfunction as well as mortality in CA [18]. Reservoir LA strain was also significantly correlated with LA-EF and end-diastolic LA area. Both, LA-EF and end-diastolic LA area are known markers of diastolic dysfunction and predictors increased mortality in patients with heart failure [19]. MRI-based LA strain analysis, therefore, needs to be evaluated as a possible predictor of mortality in patients with CA, too.

### 4.2. Differentiation between Cardiac Amyloidosis and Hypertrophic Cardiomyopathy

Differential diagnosis and clinical decision making in patients with left ventricular hypertrophy based on non-invasive imaging is challenging [20,21]. Nevertheless, the precise diagnosis of the underlying disease is crucial as different therapeutical approaches might be evoked. One relevant differential diagnosis in patients with LV hypertrophy is HCM as both CA and HCM share phenotypic features [22,23]. The mere assessment of certain characteristics, such as left ventricular wall thickness, from echocardiography or MRI is not capable to conclusively differentiate CA from HCM. The present study provides information on MRI based LAS and LASR in patients with HCM in comparison to patients with CA. Both LAS and LASR were significantly reduced in patients with HCM as compared to a healthy control group. Notably, patients with CA, LAS and LASR showed an even worse impairment compared to patients with HCM. Consequently, LAS and LASR from MRI might be a useful diagnostic parameter in patients with unclear LV hypertrophy. Larger studies are mandatory to define specific cut-off values for the differentiation of CA and HCM.

### 4.3. Clinical Outlook

Amyloidosis is associated with a decidedly poor prognosis and clinical symptoms often occur in advanced disease stages only. Early diagnosis or tools to guide suspicious echocardiographic conspicuousness into elucidation are warranted, because novel therapeutic options arrived and are under development to facilitate treatment in cardiac amyloidosis. These therapies are even more valuable the earlier a definite diagnosis is at hand, making MRI diagnostics with specifically defined characteristics widely applicable and clinically helpful. Furthermore, cardiac MRI may reduce the need of invasive endocardial biopsy as a non-invasive diagnostic tool of the differentiation of hypertrophic phenotypic mimics. This study sought to contribute to enhance the abilities of non-invasive, widely and easily available diagnosis of cardiac amyloidosis, showing the possibilities of early therapeutic strategies.

### 4.4. Limitations

The present study has an observational design that implies several limitations. The patient cohort is relatively small. Nevertheless, this study is the first to provide information on MRI-based LAS analysis in patients with biopsy-proven CA. The present cohort of patients suffered from advanced CA with a relatively high number of patients with impaired systolic LV function. Hence, conclusions may not be drawn for patients with less severe CA. Final conclusions on the performance of MRI derived left atrial strain parameters for the diagnosis of CA in patients at an early disease stage cannot be drawn from this patient cohort and warrant further studies. Furthermore, patients with CA had a lower functional status in terms of NYHA classification than patients with HCM, which might raise the question if LA strain impairment is driven more by disease severity than disease pathology. The analysis of LA LGE patterns in patients with CA and correlation to LA strain parameters may potentially clarify this problem. Further studies on LA LGE and LA strain parameters are warranted.

## 5. Conclusions

LAS and LASR are severely impaired in patients with CA. CMR strain quantification adds a further feature that can be considered too narrow down the spectrum of differential diagnosis for cardiomyopathies. Upon suspecting cardiac amyloidosis corresponding left atrial strain values may support diagnosis in combination with further clinical and radiological features. This is especially helpful in patients in whom contrast agent administration is contraindicated.

## Figures and Tables

**Table 1:** Baseline characteristics of patients with cardiac amyloidosis.

Male, *n* (%)	33 (75)
Female, *n* (%)	11 (25)
Age [years]	73.7 ± 8.9
BMI [kg/m^2^]	25.2 ± 3.5
Body surface [m^2^]	1.9± 0.2
Arterial hypertension, *n* (%)	34 (77.3)
Systolic BP [mmHg]	119.6 ± 20.9
Diastolic BP [mmHg]	74.3 ± 14.1
Heart rate [min]	72.5 ± 10.7
Left ventricular EF [%]	46.4 ± 9.6
NYHA level	
NYHA I, *n* (%)	0 (0)
NYHA II, *n* (%)	6 (13.6)
NYHA III, *n* (%)	36 (81.1)
NYHA IV, *n* (%)	2 (4.5)
NTproBNP [pg/mL]	3310 ± 897.3
Coronary artery disease, *n* (%)	14 (31.8)
Atrial fibrillation, *n* (%)	17 (38.6)
CHA2DS2-VASC Score	4 {3;5}
COPD, *n* (%)	3 (6.8)
Diabetes mellitus, *n* (%)	1 (2.3)
Stroke, *n* (%)	5 (11.4)

BMI: body mass index; BP: blood pressure; COPD: chronic obstructive pulmonary disease; EF: ejection fraction; NYHA: New York Heart Association; NTproBNP: brain natriuretic peptide.

**Table 2:** Baseline demographics of HCM patients.

Male, *n* (%)	10 (47.6)
Female, *n* (%)	11 (52.4)
Age [years]	63.9 ± 7.4
BMI [kg/m^2^]	28.3 ± 3.9
Body surface [m^2^]	1.9 ± 0.2
Arterial hypertension, *n* (%)	10 (47.6)
Systolic BP [mmHg]	129.7 ± 13.6
Diastolic BP [mmHg]	73.1 ± 9.5
Heart rate [min]	66.1 ± 7.9
Left ventricular EF [%]	53.5 ± 5.6
Maximum IVSd	20.3 ± 3.3
NYHA level	
NYHA I, *n* (%)	1 (4.8)
NYHA II, *n* (%)	8 (38.1)
NYHA III, *n* (%)	12 (57.1)
NYHA IV, *n* (%)	0
Coronary artery disease, *n* (%)	2 (9.5)
Atrial fibrillation, *n* (%)	1 (4.8)
CHA2DS2-VASC Score	2 {1;3}
COPD, *n* (%)	1 (4.8)
Diabetes mellitus, *n* (%)	3 (14.3)

**Table 3:** Baseline demographics of the healthy individuals.

Male, *n* (%)	25 (54.3)
Female, *n* (%)	21 (45.7)
Age [years]	57.3 ± 5.6
BMI [kg/m^2^]	74.8 ± 12.8
Body surface [m^2^]	1.9 ± 0.2
Left ventricular EF [%]	55 ± 1.2

**Table 4:** Histology characteristics in cardiac amyloidosis.

Histologically Proven Cardiac Amyloidosis, *n* (%)	44 (100)
ATTR, *n* (%)	22 (50)
WT, *n* (%)	22 (100)
MT, *n* (%)	0 (0)
AL, *n* (%)	22 (50)
Lambda, *n* (%)	20 (90.9)
Kappa, *n* (%)	2 (9.1)
Histological quantification	
High grade, *n* (%)	38 (86.4)
Intermediate, *n* (%)	6 (13.6)
Low grade, *n* (%)	0 (0)

ATTR: transthyretin amyloidosis; AL: light chain amyloidosis; WT: wild type; MT: mutant type.

**Table 5:** Left atrial functional parameters in ATTR and AL.

	ATTR	AL	*p*-Value
LA global longitudinal strain			
Reservoir [%]	7.9 ± 5.9	8.7 ± 4.6	0.637
Conduit [%]	5.5 ± 2.9	5.5 ± 4.6	0.988
Booster [%]	2.5 ± 4.9	3.2 ± 4.1	0.585
LA global strain rate			
Reservoir [%]	0.5 ± 0.5	0.5 ± 0.3	0.697
Conduit [%]	−0.3 ± 0.3	−0.5 ± 0.2	0.051
Booster [%]	−0.4 ± 0.4	−0.6 ± 0.4	0.104
LA function biplanar			
LA volume at LVED	86.6 ± 37	79.8 ± 32.0	0.233
LA volume at LVES	104.8 ± 39.9	102.0 ± 35.8	0.354
LA minimum volume	83.9 ± 35.3	43.6 ± 17.7	0.597
LA maximum volume	106.3 ± 40.1	103.7 ± 36.4	0.856
LA-EF	20.1 ± 10.9	26.2 ± 13.4	0.421
LA area at LVED (4CH)	23.7 ± 6.2	22.9 ± 6.1	0.139
LA area at LVES (4 CH)	27.7 ± 6.2	27.8 ± 6.3	0.163

LA: left atrial; ATTR: transthyretin amyloidosis; AL: light chain amyloidosis; LVED: left ventricular end diastole; LVES: left ventricular end systole; CH: chamber.

**Table 6:** Pearson’s correlation for left atrial reservoir strain and other disease-relevant factors in cardiac amyloidosis.

	Pearson’s Correlation	*p*-Value
Global LA reservoir strain		
LV strain	0.445	0.002
LA area (LVED)	0.374	0.012
LA-EF	0.518	<0.001
LV-EF	0.365	0.015
Global LA conduit strain		
Global longitudinal LV strain	0.500	<0.001
LA-EF	0.483	<0.001
Global LA reservoir strain rate		
LV strain	0.318	0.036
LA-EF	0.335	0.026
LV-EF	0.319	0.035
Global LA conduit strain rate		
LV strain	0.318	0.035
Global LA booster strain rate		
LV-EF	0.311	0.04
LA-EF	0.576	<0.001
LA area (LVED)	0.531	<0.001
LA area (LVES)	0.39	0.009

LA: left atrial; LA-EF: left atrial ejection fraction; LV-EF: left ventricular ejection fraction; ATTR: transthyretin amyloidosis; AL: light chain amyloidosis; LVED: left ventricular end diastole; LVES: left ventricular end systole.

## Data Availability

The data underlying this article will be shared upon reasonable request to the corresponding author.
